# Lysophosphatidic acid receptor 6 regulated by miR-27a-3p attenuates tumor proliferation in breast cancer

**DOI:** 10.1007/s12094-021-02704-8

**Published:** 2021-09-12

**Authors:** J. Lei, S. Guo, K. Li, J. Tian, B. Zong, T. Ai, Y. Peng, Y. Zhang, S. Liu

**Affiliations:** 1 Endocrine Breast Surgery, The First Affiliated Hospital of Chongqing Medical University, No.1 Youyi Road, Yuzhong District, Chongqing, 400016 China; 2 Department of Cardiology, Chongqing Kanghua Zhonglian Cardiovascular Hospital, Jiangbei District, No. 168 Haier Rd, Chongqing, 400016 China

**Keywords:** Lysophosphatidic acid receptor 6, miR-27a-3p, Breast cancer, Cell proliferation

## Abstract

**Purpose:**

Lysophosphatidic acid (LPA) is a bioactive molecule which participates in many physical and pathological processes. Although LPA receptor 6 (LPAR6), the last identified LPA receptor, has been reported to have diverse effects in multiple cancers, including breast cancer, its effects and functioning mechanisms are not fully known.

**Methods:**

Multiple public databases were used to investigate the mRNA expression of LPAR6, its prognostic value, and potential mechanisms in breast cancer. Western blotting was performed to validate the differential expression of LPAR6 in breast cancer tissues and their adjacent tissues. Furthermore, in vitro experiments were used to explore the effects of LPAR6 on breast cancer. Additionally, TargetScan and miRWalk were used to identify potential upstream regulating miRNAs and validated the relationship between miR-27a-3p and LPAR6 via real-time polymerase chain reaction and an in vitro rescue assay.

**Results:**

LPAR6 was significantly downregulated in breast cancer at transcriptional and translational levels. Decreased LPAR6 expression in breast cancer is significantly correlated with poor overall survival, disease-free survival, and distal metastasis-free survival, particularly for hormone receptor-positive patients, regardless of lymph node metastatic status. In vitro gain and loss-of-function assays indicated that LPAR6 attenuated breast cancer cell proliferation. The analyses of TCGA and METABRIC datasets revealed that LPAR6 may regulate the cell cycle signal pathway. Furthermore, the expression of LPAR6 could be positively regulated by miR-27a-3p. The knockdown of miR-27a-3p increased cell proliferation, and ectopic expression of LPAR6 could partly rescue this phenotype.

**Conclusion:**

LPAR6 acts as a tumor suppressor in breast cancer and is positively regulated by miR-27a-3p.

**Supplementary Information:**

The online version contains supplementary material available at 10.1007/s12094-021-02704-8.

## Introduction

Breast cancer accounts for 30% of the estimated incidence amongst all cancers in females and 15% of the estimated cancer-related deaths worldwide [1]. With improved early diagnosis and treatments, the total breast cancer-associated mortality in females has dropped by 31% [1]. However, resistance to endocrine therapy and chemotherapy in patients with breast cancer typically leads to regional recurrence and distal metastasis, which causes high mortality [1, 2]. Notably, breast cancer exhibits high heterogeneity [2, 3], particularly, intratumoral heterogeneity, which is generated from both extrinsic factors from the tumor microenvironment and intrinsic parameters from the cancer cells [4]. The intrinsic parameters primarily include genetic, epigenetic, and transcriptomic traits, which affect gene expression and activation of related pathways. This heterogeneity affects the effectiveness of treatments; therefore, novel targets for precision therapies must be identified.

Lysophosphatidic acid (LPA) is a bioactive molecule which participates in many physical and pathological processes, such as brain development, pain, asthma, heart disease, and cancer [5–9]. In cancers, LPA functions as a procancerous substance which, together with corresponding receptors, induces cancer cell proliferation, migration, invasion, angiogenesis, inflammation, and other effects [10, 11]. However, there are studies with contradictory findings [12–14]; hence, more studies are needed to ascertain the roles of LPA in cancer with regard to its receptors. There are six types of LPA receptors (LPARs; LPAR1-6) which belong to G protein-coupled receptors (GPCRs), characterized by seven transmembrane helices. LPAR1-5 have been well documented; however, LPAR6 is relatively poorly studied [11, 15]. LPAR6 was first reported in hypotrichosis simplex [16] and afterwards was implicated in the initiation and progression of cancer [13, 14, 17–22]. LPAR6 functions to reduce intestinal cell adhesion through binding to specific GPCRs, Gαi, or Gα12/13 and regulates downstream extracellular signal-regulated kinase 1/2 (ERK1/2) and Rho/Rho-associated kinase (ROCK) pathways [15]. However, the biological functions and regulatory mechanisms of LPAR6, particularly its relationship with microRNA, in breast cancer are unclear and need further research.

Non-coding RNAs (ncRNAs) are a class of molecules which play important roles in regulating cellular activity [23]. MicroRNAs (miRNAs) are short ncRNAs with approximately 22 nucleotides in length and act as oncogenes or suppressors for cancers through targeting specific mRNAs [23]. For instance, the small ncRNA, miR-27a-3p, located on chromosome 19, acts as an oncogenic RNA in renal clear cell carcinoma, gastric cancer, breast cancer, and colorectal cancer [24, 25]. Additionally, miR-27a-3p participates in drug resistance in leukemia, ovarian cancer, and hepatocellular carcinoma [26]. However, miR-27a-3p also has antitumor effects in non-small cell lung cancer [27]. Although miR-27a-3p has been reported to promote triple-negative breast cancer (TNBC) progression via targeting glycogen synthase kinase 3β (GSK3β) or v-Akt murine thymoma viral oncogene/protein kinase-B (Akt) [28, 29], it is not fully investigated in non-TNBC.

In this study, the functions and regulatory mechanisms of LPAR6 in breast cancer were investigated further. We demonstrated that LPAR6 acts as a tumor suppressor in breast cancer and that miR-27a-3p positively regulated LPAR6 expression and, hence, attenuated cell proliferation in breast cancer. Therefore, the miR-27a-3p/LPAR6 axis would be a potential target for the therapeutic strategy of breast cancer.

## Materials and methods

### Cell culture and transfection

Human breast cancer cell lines (MCF-7, ATCC number HTB-22; ZR-75–1, ATCC number CRL-1500; T47D, ATCC number HTB-133; SK-BR-3, ATCC number HTB-30; BT549, ATCC number HTB-122; MDA-MB231, ATCC number HTB-26; MDA-MB436, ATCC number HTB-130; and MDA-MB468, ATCC number HTB-132) and a normal mammary epithelial cell line (MCF-10A, ATCC number CRL-10317) were obtained from the American Type Culture Collection (ATCC). All breast cancer cell lines were maintained in Dulbecco’s modified Eagle medium (DMEM; Gibco, Grand Island, NY, USA) supplemented with 10% fetal bovine serum (FBS; Gibco). MCF-10A cell line was maintained in a special medium (cat# CM-0525; Procell, Wuhan, China). All cell lines were cultured at 37 ℃ in a humidified incubator with 5% CO_2_. Cells were seeded in 6-well plates and cultured for approximately 24 h and then transfected with corresponding plasmids, small interfering RNAs (siRNAs), or miRNA mimics or inhibitor when the cell confluence reached 80–90%.

### Sample collection

Breast cancer samples were collected in the First Affiliated Hospital of Chongqing Medical University from August to September 2019. Written informed consent forms were signed by patients prior to surgical operations. All the procedures were approved by the Ethics Committee of the First Affiliated Hospital of Chongqing Medical University and performed according to the ethical standards laid down in the 1964 Declaration of Helsinki and its subsequent amendments.

### Overexpressing adenovirus, siRNAs, microRNA mimics, and inhibitor

Three siRNAs targeting LPAR6 mRNA and negative control (NC) siRNA were purchased from GenePharma (Shanghai, China). Adenoviral vectors which can overexpress the LPAR6 coding sequence (CDS) were purchased from Hanbio (Shanghai, China). The miR-27a-3p mimics, inhibitor, and their corresponding control oligonucleotides were synthesized by GenePharma. The sequences of the oligonucleotides mentioned above were listed in Table 1.

**Table 1:** Sequences of LPAR6 siRNA, miR-27a-3p mimics, inhibitor and their corresponding control oligonucleotides

Gene		Orientation	Sequence (5’ → 3’)
LPAR6	siRNA NC	Sense	UUCUCCGAACGUGUCACGUTT
		Antisense	ACGUGACACGUUCGGAGAATT
	si-1	Sense	GCUCCCACUGCUUCUAUAATT
		Antisense	UUAUAGAAGCAGUGGGAGCTT
	si-2	Sense	GGUGUUUGUGCUUGGGUUATT
		Antisense	UAACCCAAGCACAAACACCTT
	si-3	Sense	GCAUAACCUACAGACCUUATT
		Antisense	UAAGGUCUGUAGGUUAUGCTT
miR-27a-3p	Mimics NC	Sense	UUCUCCGAACGUGUCACGUTT
		Antisense	ACGUGACACGUUCGGAGAATT
	Mimics	Sense	UUCACAGUGGCUAAGUUCCGC
		Antisense	GGAACUUAGCCACUGUGAAUU
	Inhibitor NC		CAGUACUUUUGUGUAGUACAA
	Inhibitor		GCGGAACUUAGCCACUGUGAA

### Cell proliferation assay

Cells were seeded at a density of 3000–5000 cells per well in 96-well plates and maintained with DMEM supplemented with 10% FBS. The cell proliferation assay was performed using a Cell Counting Kit 8 (CCK8; MCE, Monmouth Junction, NJ, USA). After treatment with CCK8, cells were continuously cultured in an incubator for 2 h without light, and then, optical density was measured using a microplate reader (BioTek, Winooski, VT, USA) at 450 nm.

### Cell plate colony formation assay

Cells were seeded at a density of 500–1000 cells per well in 6-well plates in DMEM containing 10% FBS and cultured for approximately 10–14 days. The medium was replaced every 2 days. When cell colonies were detectable with naked eyes, cells were washed twice with precooled phosphate-buffered saline (PBS), fixed with 4% paraformaldehyde for 30 min, and washed twice again with PBS. Subsequently, cell colonies were stained with 0.5% crystal violet solution for 30 min. The colonies were counted using Image J 1.52a software (National Institutes of Health, Bethesda, MD, USA).

### Real-time quantitative polymerase chain reaction (RT-qPCR)

Total RNA and microRNA were isolated using the Simply P Total RNA Extraction Kit and microRNA Extraction Kit (BioFlux, Hangzhou, China), respectively. The concentration and A260/280 ratio of total RNA were determined by NanoDrop™ 2000c spectrophotometer (Thermo Fisher Scientific, Waltham, MA, USA) for quality control. Complementary DNA (cDNA) synthesis was performed using 1 μg of the total RNA with the PrimeScript™ II cDNA Synthesis Kit (TaKaRa, Shiga, Japan). RT-qPCR was performed using predesigned primers and SYBR Premix Ex Taq™ II (TaKaRa) with a CFX96 Touch™ Fluorescence Quantitative PCR instrument (Bio-Rad, Hercules, CA, USA). The primers used in this study are listed in Table 2. GAPDH, β-actin, and U6 were used as internal controls for LPAR6 and miR-27a-3p. The primers were all obtained from Sangon Biotech (Shanghai, China). The 2^−△△Ct^ method was used to calculate the fold change of target gene expression.

**Table 2:** Primers used in this study

Gene	Accession number	Primer	Sequence (5’ → 3’)
LPAR6	NM_001162497	Forward	TTTGCACTGGCGTGTGGTT
		Reverse	TCTGAGGCATTGTTACCCTGA
GAPDH	NM_002046	Forward	CTCTGCTCCTCCTGTTCGAC
		Reverse	GCGCCCAATACGACCAAATC
β-actin	NM_001101.5	Forward	CATGTACGTTGCTATCCAGGC
		Reverse	CTCCTTAATGTCACGCACGAT
miR-27a-3p	MIMAT0021906	Reverse Transcription primer	GTCGTATCCAGTGCAGGGTCCG AGGTATTCGCACTGGATACGACG CGGAA
		Forward	GCGCGTTCACAGTGGCTAAG
		Reverse	AGTGCAGGGTCCGAGGTATT
U6	NR_004394	Reverse transcription primer	AACGCTTCACGAATTTGCGT
		Forward	CTCGCTTCGGCAGCACA
		Reverse	AACGCTTCACGAATTTGCGT

### Protein extraction and western blotting

Breast cancer cell lines and tissues were lysed using radioimmunoprecipitation assay buffer (Solarbio, Beijing, China) and protease inhibitor (cat# HY-K0011; MCE) on ice, and the protein concentrations were measured with a BCA Protein Assay Kit (Bio-Rad). Western blotting was performed using an electrophoresis apparatus (Bio-Rad). Briefly, protein samples were loaded on sodium dodecyl sulfate–polyacrylamide gels, followed by electrophoresis for approximately 2 h, and then transferred to a polyvinylidene difluoride membrane. After blocking with 5% (w/v) fat-free milk for 1.5 h at room temperature, the membrane was incubated with the corresponding primary antibodies followed by incubation with the appropriate horseradish peroxidase-conjugated secondary antibodies and imaging with electrochemiluminescence. Immunoreactive bands were detected using an automatic Genesys Imager (Bio-Rad). The primary and secondary antibodies used are listed below: anti-LPAR6 antibody (cat# AP52517PU-N; OriGene, Rockville, MD, USA) and anti-GAPDH antibody (cat# 10,494–1-AP; Proteintech, Wuhan, China); and anti-mouse secondary antibody (cat# SA00001-1; Proteintech) and anti-rabbit secondary antibody (cat# SA00001-15; Proteintech), respectively. The anti-GAPDH antibody was the internal control.

### Bioinformatics analysis

Data from The Cancer Genome Atlas (TCGA) Program and molecular taxonomy of breast cancer international consortium (METABRIC) were collected to perform differential expression and pathway analyses. The UALCAN server (http://ualcan.path.uab.edu/index.html) was used to investigate the LPAR6 expression in breast cancer subtypes [30], and the breast cancer gene-expression miner v4.6 (bc-GenExMiner v4.6; http://bcgenex.ico.unicancer.fr) was used to perform survival analysis [31]. The gene set enrichment analysis (GSEA) was performed using the clusterProfiler package [32] in R4.0.3. Furthermore, hallmark gene sets were downloaded from the Msigdb homepage (https://www.gsea-msigdb.org/gsea/index.jsp). To predict upstream miRNAs which may regulate LPAR6 expression, online miRWalk (http://mirwalk.umm.uni-heidelberg.de/) [33] and TargetScan (http://www.targetscan.org/vert_72/) [34] were utilized.

### Statistical analysis

GraphPad Prism 8.0.1 and R 4.0.3 were used for data analysis and visualization. The *t *test and one-way ANOVA were used to determine the significance between two groups and among several groups, respectively. Data are presented as mean ± SD. Correlation between LPAR6 and other protein-coding genes was assessed using the Pearson method in R. All experiments were repeated at least three times, and *p* < 0.05 was considered statistically significant. (*p < 0.05, **p < 0.01, ***p < 0.001, ****p < 0.0001).

## Results

### LPAR6 is downregulated in breast cancer, and decreased LPAR6 expression is correlated with poor clinicopathological features

**Fig. 1:**
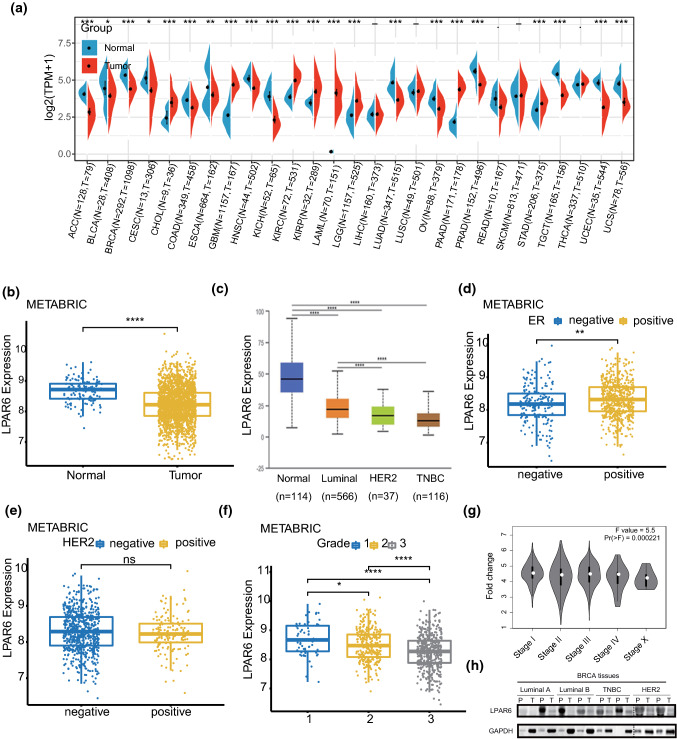
LPAR6 is downregulated in breast cancer, and decreased LPAR6 expression is correlated with poor clinicopathological features. **a** Different LPAR6 mRNA expression levels were determined from pan-cancer data (data from TCGA). **b** Differential LPAR6 mRNA expression in normal tissue and tumors from METABRIC dataset. ****p < 0.0001 (*t* test). **c** LPAR6 mRNA expression in normal tissues and tissues of subtypes of breast cancer (UALCAN). ****p < 0.0001 (*t* test). **d** LPAR6 mRNA expression in ER-positive and -negative breast cancer. **p < 0.01 (*t* test). **e** LPAR6 mRNA expression in HER2-positive and -negative breast cancer. ns: not significant (*t* test). **f** LPAR6 mRNA expression in three pathological grades of breast cancer. *p < 0.05, ****p < 0.0001 (Kruskal–Wallis test). **g** LPAR6 mRNA expression in clinical stages of breast cancer. **h** LPAR6 expression in tissues of four subtypes of breast cancer assessed using western blotting. GAPDH was used as an internal control. Abbreviations: *ACC* adrenocortical carcinoma, *BLCA* bladder urothelial carcinoma, *BRCA* breast invasive carcinoma, *CESC* cervical squamous cell carcinoma and endocervical adenocarcinoma; *CHOL* cholangiocarcinoma, *COAD* colon adenocarcinoma, *ER* estrogen receptor, *ESCA* esophageal carcinoma, *GAPDH* glyceraldehyde-3-phosphate dehydrogenase, *GBM* glioblastoma multiforme, *HER2* human epidermal growth factor receptor 2, *HNSC* head and neck squamous cell carcinoma, *KICH* kidney chromophobe, *KIRC* kidney renal clear cell carcinoma, *LAML* acute myeloid leukemia, *LGG* brain lower grade glioma, *LIHC* liver hepatocellular carcinoma, *LUAD* lung adenocarcinoma, *LUSC* lung squamous cell carcinoma, *METABRIC* molecular taxonomy of breast cancer international consortium, *OV* ovarian serous cystadenocarcinoma, *P* para-cancer tissues, *PAAD* pancreatic adenocarcinoma, *PRAD* prostate adenocarcinoma, *READ* rectum adenocarcinoma, *SKCM* skin cutaneous melanoma, *STAD* stomach adenocarcinoma, *T* paired tumor tissues, *TCGA* The Cancer Genome Atlas, *TGCT* testicular germ cell tumors, *THCA* thyroid carcinoma, *TNBC* triple-negative breast cancer, *UCEC* uterine corpus endometrial carcinoma, *UCS* uterine carcinosarcoma

To determine LPAR6 expression in different cancer types, pan-cancer data from TCGA were analyzed, and the results showed that LPAR6 was differentially expressed in cancer types (Fig. 1a). By exploring the METABRIC dataset, LPAR6 was significantly downregulated in breast cancer tissues compared with that in normal controls (Fig. 1b). Additionally, LPAR6 expression was significantly higher in the luminal subtype than that in the human epidermal growth factor receptor 2 (HER2) and TNBC subtypes (Fig. 1c) using UALCAN database. Estrogen receptor (ER)-positive patients exhibited significantly increased LPAR6 expression level compared with ER-negative patients in METABRIC dataset, which was consistent with the results from UALCAN (Fig. 1c and d). However, there was no significant difference in LPAR6 expression between HER2-positive and -negative patients (Fig. 1e). Interestingly, patients with a higher pathological grade or clinical stage had significantly lower LPAR6 expression (Fig. 1f and g). Notably, LPAR6 was downregulated in breast cancer tissues among all ethnicities, particularly in African American and Asian groups, compared with Caucasians (Fig. S1a). Meanwhile, there were no significant differences in LPAR6 expression in patients with breast cancer with different lymphatic statuses (Fig. S1b). To corroborate the above findings, a western blot assay was performed, and the results showed that LPAR6 protein level was lower in the para-tumor group than that in the tumor group (Fig. 1h).

Genetic and epigenetic alterations lead to gene expression alterations at transcriptional and post-transcriptional levels. For example, a previous study reported that CpG islands of LPAR6 were significantly hypermethylated [13]. To determine whether genetic alterations participated in the dysregulated expression of LPAR6, we explored the cBioPortal for cancer genomics database [35] and catalog of somatic mutations in cancer (COSMIC) database (https://cancer.sanger.ac.uk/cosmic). The results showed that genetic alteration of LPAR6 accounted for 6% of all the samples (Fig. S1c). Among these altered samples, genetic deep deletion and other factors causing low mRNA levels were the major genetic alterations (Fig. S1c). Considering that different genetic alterations leads to different LPAR6 expression levels, deep deletion significantly decreased the expression of LPAR6 compared with other types of genetic alterations (Fig. S1d). Regarding mutation type, missense substitutions (15.62%) were the major mutation type of LPAR6 in all the samples, and C > T (50%) was the major base mutation type (Fig. S1e). However, survival analysis of the genetic alteration revealed that it had no significant effects on patient overall survival (OS) and relapse-free survival (Fig. S1f and g). This is possibly because the proportion of LPAR6 genetic alterations accounting for dysregulated expression of LPAR6 was extremely low to affect the prognosis. Overall, LPAR6 expression is downregulated in breast cancer, and this is correlated with poor clinicopathological features, including pathological grades and clinical stages, which indicates that LPAR6 acts as a suppressor in breast cancer. Additionally, genetic alterations may not be the main factor attributed to LPAR6 dysregulated expression.

### Decreased LPAR6 expression is significantly correlated with poor survival especially for hormone receptor-positive (HR +) patients in breast cancer

**Fig. 2:**
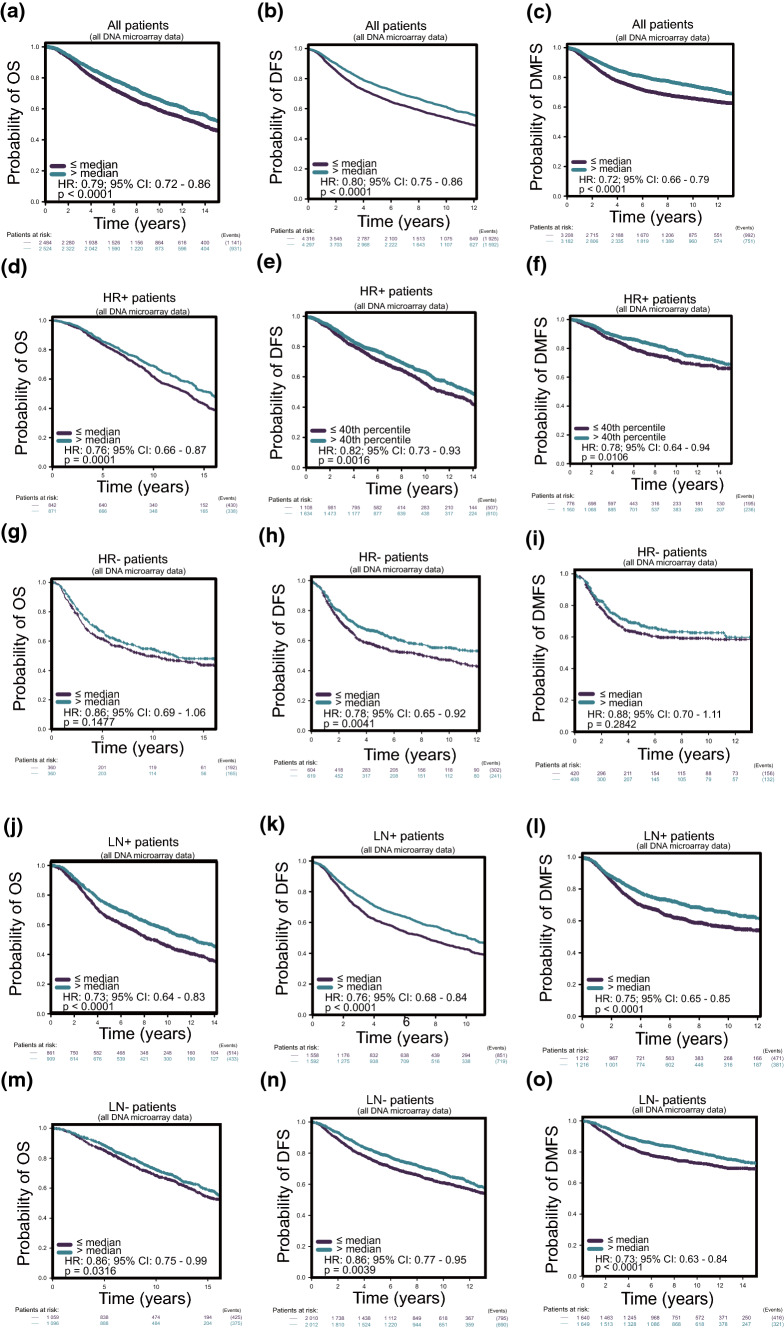
Decreased LPAR6 expression in breast cancer is significantly correlated with poor survival especially for hormone receptor-positive (HR +) patients. **a–c** Decreased LPAR6 expression significantly predicted poor overall survival (OS), disease-free survival (DFS), and distal metastasis-free survival (DMFS) in all patients. **d–f** Decreased LPAR6 expression significantly predicted poor OS, DFS, and DMFS in (HR +) patients. **g–i** Decreased LPAR6 expression could not predict OS and DMFS in HR- patients well but could predict DFS. **j–o** Decreased LPAR6 expression significantly predicted poor OS, DFS, and DMFS in patients with positive or negative lymph node metastasis

The bc-GenExMiner v4.6 was used to analyze the prognostic value of LPAR6 expression in breast cancer [31]. The results showed that decreased LPAR6 expression in all patients with breast cancer was significantly correlated with poorer OS, disease-free survival (DFS), and distal metastasis-free survival (DMFS) compared with that in patients with high LPAR6 expression (Fig. 2a–c). Moreover, subgroup survival analyses revealed that, in HR + patients, the decreased LPAR6 expression was also related to unfavorable OS, DFS, and DMFS (Fig. 2d–f). However, there were no significant differences in OS, DFS, and DMFS between low and high LPAR6 expression in HR negative (HR-) patients (Fig. 2g–i). Interestingly, LPAR6 expression was significantly correlated with patient survival regardless of the lymph node (LN) metastatic status (Fig. 2j–o). Overall, the LPAR6 expression level is significantly correlated with prognosis in all patients with breast cancer, even in HR + patients, regardless of LN metastatic status.

### LPAR6 inhibits breast cancer cell proliferation in vitro

**Fig. 3:**
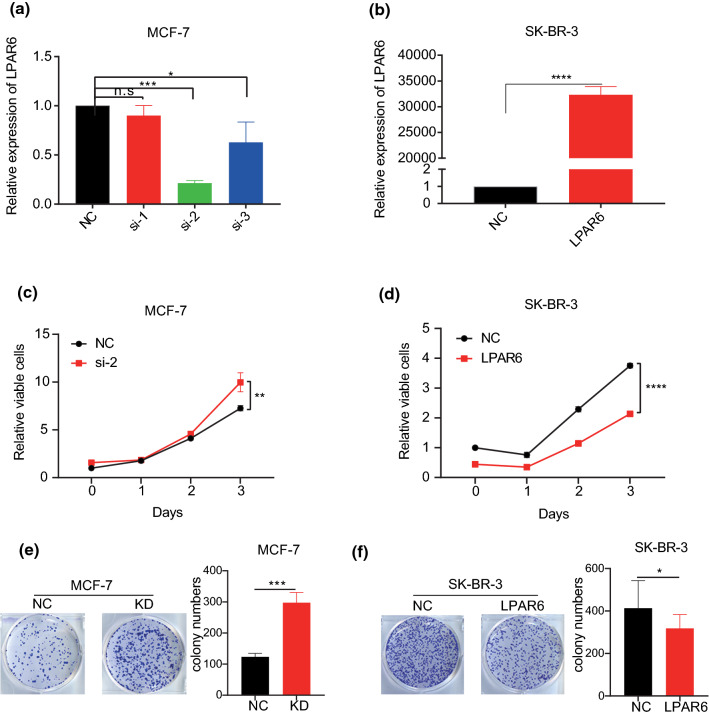
LPAR6 inhibits breast cancer cell proliferation in vitro. **a** Efficient knockdown (KD) of LPAR6 with siLPAR6 in MCF-7 cells. *p < 0.05; ****p < 0.001; *ns* not significant (*t* test). **b** Efficient overexpression (OE) of LPAR6 with adenovirus in SK-BR-3 cells. ****p < 0.001 (*t* test). **c** and **d** Cell counting kit-8 assay was performed in MCF-7 and SK-BR-3 cells. **p < 0.01, ****p < 0.0001 (*t* test). **e** and **f** Plate colony formation assay was performed in MCF-7 and SK-BR-3 cells. *p < 0.05; ***p < 0.001 (*t* test)

To determine the biological functions of LPAR6 in breast cancer, we performed knockdown and overexpression assays of LPAR6 (Fig. 3a and b, Table S1 and S2). As siLPAR6-2 (si-2) reached the best knockdown efficiency (approximately 70%) (Fig. 3a), it was selected to perform further experiments. As shown in Fig. 3c, LPAR6 knockdown significantly increased viability in the MCF-7 cells compared with the NC group. Alternatively, ectopic expression of LPAR6 inhibited viability in SK-BR-3 cells compared with that of the pcDNA3.1 empty vector-transfected (NC) cells (Fig. 3d). Furthermore, LPAR6 knockdown significantly increased colony numbers in MCF-7 cells (Fig. 3e), and ectopic expression of LPAR6 decreased colony numbers in SK-BR-3 cells (Fig. 3f). These results showed that LPAR6 can inhibit cell proliferation. Taken together, LPAR6 inhibits breast cancer growth via attenuating cell proliferation and acts as a tumor suppressor in breast cancer.

### Bioinformatics analyses of TCGA and METABRIC datasets show that LPAR6 may be involved in the cell cycle arrest pathway

To investigate LPAR6 effects on the inhibition of breast cancer progression, we investigated its significantly correlated or co-expressed genes via in silico analysis. An alternative approach is the concept of “guilt-by-association” (GBA) which assumes that if two proteins interact or share expression patterns, their functions are more likely to be related [36, 37]. To reduce the influence of confounding factors, we sorted samples according to the expression level of LPAR6 and selected the first 200 and the last 200 samples to constitute two groups: “high”-level group and “low”-level group both in TCGA and METABRIC datasets, respectively.

**Fig. 4:**
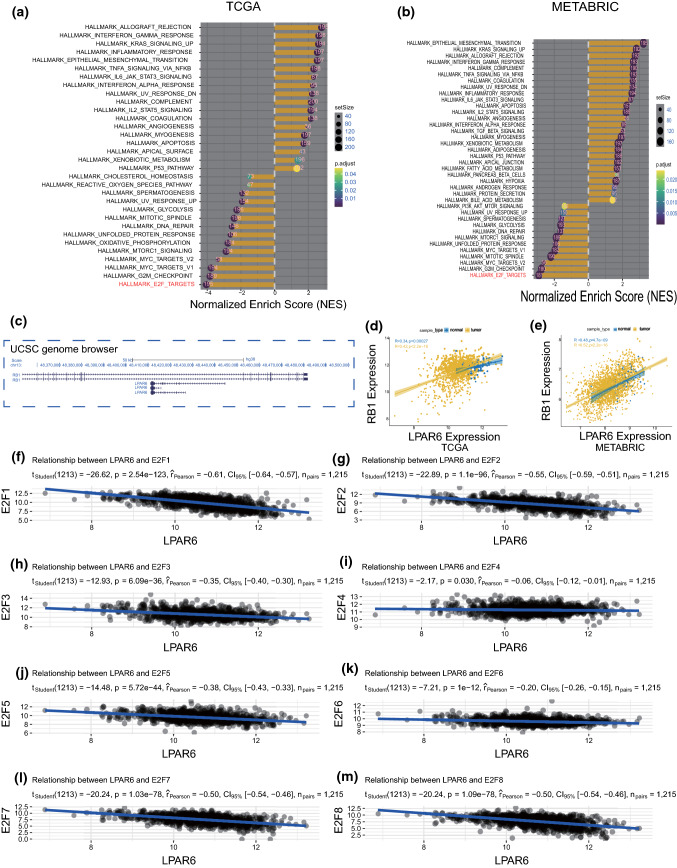
TCGA and METABRIC data analyses show that LPAR6 may be involved in the cell cycle arrest pathway. **a** and **b** GSEA analyses using TCGA and METABRIC datasets (red word indicating the most enriched pathway in both datasets). **c** Visualization of LPAR6 and RB1 genomic location using UCSC genome browser. **d** and **e** Pearson correlation between LPAR6 and RB1 expression in normal tissues and tumor groups of TCGA and METABRIC datasets, respectively. **f–m** Pearson correlation between LPAR6 expression and expression of E2F family members in tumors of TCGA dataset

Next, we performed the Pearson correlation test between LPAR6 and the other genes and selected statistically significant genes (*p* < 0.05) to undergo further analysis. Correlation values of genes were ranked, and GSEA was performed. Interestingly, hallmark E2F, G2/M checkpoint, and myc target pathways were all suppressed, and this corresponded with the results from TCGA and METABRIC datasets (Fig. 4a and b). Thus, we inferred that LPAR6 inhibits cell proliferation, and this may be mediated through cell cycle arrest, as demonstrated in our previous work [13].

As the E2F family is well-characterized in the cell cycle process [38–41], we paid attention to the mechanisms of E2F-regulated cell cycle arrest. The E2F1-3 proteins bind to retinoblastoma protein (RB1) to regulate cell cycle progression [40]. Considering that a target gene may function similarly to its neighbors in the genome [42], we searched the genomic location of *LPAR6* and *RB1* in the UCSC genome browser. As shown in Fig. 4c, *LPAR6* was located within *RB1* in the reverse orientation. Correlation analyses also supported that LPAR6 and RB1 shared similar expression patterns both in healthy breast tissues and breast cancer tissues (Fig. 4d and e). Further correlation analyses between LPAR6 and E2F family members revealed that LPAR6 was significantly related to the E2F family, particularly E2F2 (Fig. 4f–m, Fig. S2). Taken together, LPAR6 may induce cell cycle arrest to exhibit antitumor effects via interacting with the RB1/E2F family complexes.

### LPAR6 is positively regulated by miR-27a-3p in breast cancer

**Fig. 5:**
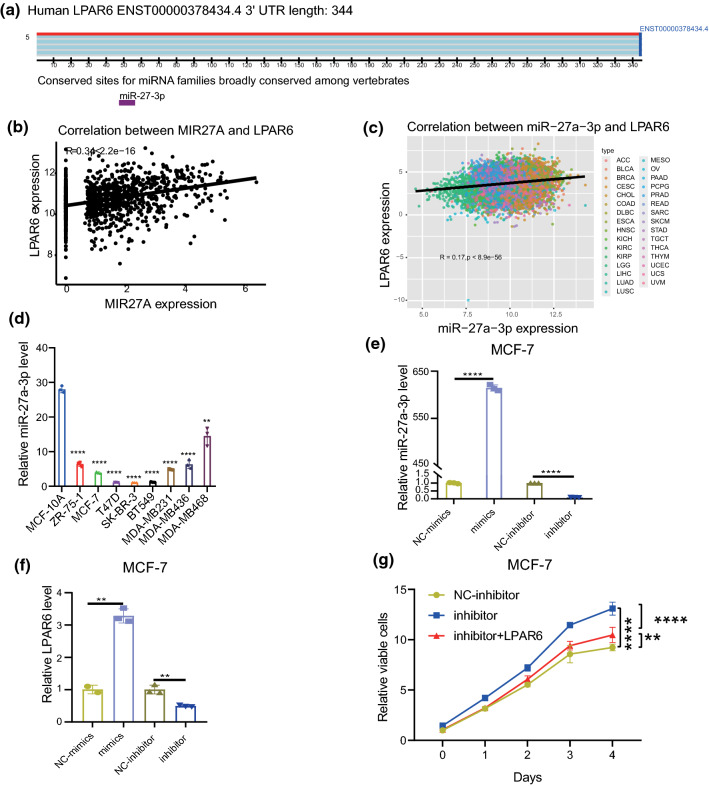
LPAR6 is positively regulated by miR-27a-3p in breast cancer. **a** Predicted binding site of miR-27a-3p on LPAR6 3′ untranslated region (3′ UTR) via TargetScan tool. The purple square indicates miR-27a-3p. **b** Pearson correlation of LPAR6 and miR-27a-3p precursor MIR27A expression. Data were from TCGA dataset. **c** Pearson correlation of LPAR6 and mature miR-27a-3p expression. Data were from TCGA dataset. **d** miR-27a-3p expression in a healthy mammary epithelial cell line (MCF-10A) and breast cancer cell lines. **p < 0.01, ****p < 0.0001 (compared to MCF-10A, *t* test). **e** Validation of miR-27a-3p manipulation with miRNA mimics and inhibitor. ****p < 0.0001 (t test). **f** LPAR6 mRNA level was regulated by miR-27a-3p expression. **p < 0.01 (*t* test). **g** Cell counting kit-8 assay showed deregulation of miR-27a-3p can increase cell proliferation, and LPAR6 ectopic expression can partly rescue this phenotype. **p < 0.01, ****p < 0.0001 (compared to negative control, *t* test)

Although protein-coding genes play important roles in cancer biological processes, ncRNAs also have key regulatory roles in shaping the activity of cancer cells [23]. To uncover how LPAR6 is regulated by miRNAs, we searched potential miRNAs via TargetScan and miRWalk. Notably, hsa-miR-27a-3p was reported on both servers. TargetScan results showed that miR-27a-3p binds to the 3′ untranslated region (3′ UTR) of *LPAR6* (Fig. 5a), and miRWalk analysis revealed that miR-27a-3p binds to the CDS of *LPAR6* (data not shown). To verify a possible regulatory relationship, we initially performed correlation analysis between miR-27a-3p and LPAR6 using TCGA dataset. Interestingly, both pri-miRNA and mature miRNA of miR-27a-3p positively correlated with LPAR6 (Fig. 5b and c), which was different from the well-known canonical function of miRNAs. Notably, miRNAs may regulate target genes by binding with the 5′ UTR and CDS [43]. From the above results, we postulated that miR-27a-3p may positively regulate LPAR6 transcription via binding to the CDS of LPAR6.

To further verify this regulation, we determined the expression of miR-27a-3p in human mammary epithelial cell and breast cancer cell lines (Fig. 5d, Table S3). Subsequently, the MCF-7 cell line was selected to be transfected with miR-27a-3p mimics, inhibitor, and corresponding NC oligonucleotides. The transfection efficiency of miR-27a-3p mimics and inhibitor was validated using real-time PCR (Fig. 5e, Table S4). Consistent with our hypothesis, miR-27a-3p mimics upregulated LPAR6 mRNA levels, and the miR-27a-3p inhibitor decreased the expression of LPAR6 mRNA (Fig. 5f, Table S5). Moreover, the proliferation assay revealed that miR-27a-3p knockdown increased growth in the MCF-7 cells, and LPAR6 overexpression partly rescued this phenotype (Fig. 5g). Taken together, the results demonstrate that miR-27a-3p positively regulates LPAR6 mRNA levels and attenuates cancer cell proliferation via LPAR6 in breast cancer.

## Discussion

Breast cancer is the most common cancer and the second-leading cause of cancer-related deaths among women [44, 45]. Though the mortality of breast cancer has decreased, many female patients suffer because it may lead to adverse drug reactions and mental anxiety and may require surgery [1, 46]. Importantly, more effective therapies are urgently needed.

GPCRs account for 34% of small molecular drug targets in diseases [47]. As a sub-family of GPCRs, LPAR6 has the potential to be targeted for disease therapy; it exhibits different roles in different organs affected by cancer [12, 13, 17–19, 21, 22]. Our results also support its diverse, even opposite, functions in different cancers (Fig. 1a). As an oncogene, LPAR6 expression is increased in tumors compared with that in para-tumors or normal tissues. It promotes cancer initiation and progression and enhances cancer cell motility, invasion, and colony formation in liver cancer, pancreatic cancer, ovarian cancer, and prostate cancer [12, 19, 22]. In contrast, LPAR6 may act as an antitumor factor and inhibit cancer cell motility in colon cancer [14], and this antitumor role may exist in breast cancer [13]. Our study validates the tumor suppressor role of LPAR6 through in vitro experiments of representative cell lines of luminal and HER2 subtypes of breast cancer.

To validate the clinical effects of LPAR6 in breast cancer, we analyzed its prognostic value. Consistent with the results of in vitro experiments and bioinformatics analyses, patients with high LPAR6 level were demonstrated to have a good prognosis, thereby providing further evidence for its role as a tumor suppressor. Decreased LPAR6 expression exhibited a poor prognosis in OS, DFS, and DMFS in all patients, which was also determined to be true in HR + subtypes and LN metastatic positive or negative subtypes. To date, research on LPAR6 in breast cancer is relatively rare [11]. Although previous studies have suggested that LPAR6 served as an oncogene in hepatocellular carcinoma and pancreatic carcinoma, it is rational to consider, from our results, that in breast cancer, LPAR6 acts as an antitumor factor. A rational explanation is that the biological function and underlying mechanisms of specific proteins are cell context-dependent.

Previous studies have suggested that LPAR6 affects tumor biological functions through Gα12/13-Rho, adenylyl cyclase (AC)/cyclic adenosine monophosphate-dependent/protein kinase A (PKA), Ca^2+^-protein kinase C (PKC) pathways, and that it is regulated by nuclear receptor coactivator 3 (NCOA3) [10, 22]. To realize other potential mechanisms underlying the effects of LPAR6 on breast cancer, we performed GSEA using TCGA and METABRIC datasets. Interestingly, bioinformatics analyses of the two large datasets revealed that hallmark E2F, G2/M checkpoint, and myc target pathways were all significantly suppressed with regard to LPAR6 in breast cancer (Fig. 4a and b). The results are consistent with the phenotypes of experiments in vitro (Fig. 3c-f) and support our findings. Additionally, the results from GBA method predict other possible functions of LPAR6 in breast cancer, indicating several convincible directions for future research.

It is established that GPCRs function through binding to Gα subunit (e.g., Gαs, Gαi/o, Gα12/13, and Gαq/11) to activate or inactivate downstream signals (phospholipase C, AC, phosphoinositide 3-kinases) to promote or inhibit tumor progression. Interestingly, signals through Gαs subunit can activate AC and PKA, thereby phosphorylating the large tumor suppressor 1 and 2 (LATS1/2), which exerts antitumor effects in medulloblastoma and basal cell carcinoma [48, 49]. Therefore, we inferred that LPAR6 may inhibit breast cancer growth via activating Gαs-AC-PKA-Hippo pathway, which is supported by a recent review [11].

MicroRNAs exhibit diverse roles in the molecular and cellular processes of cancer [23]. As a classical function, miRNAs downregulate target gene expression at post-transcriptional and translational levels by binding to the 3′ UTR of the gene. Notably, miRNAs also upregulate target gene expression by binding to their promoter or CDS regions, which serve as an unconventional regulatory mechanism [43, 50, 51]. MiR-27a-3p, a not fully investigated miRNA, has been reported to have controversial functions in cancers. For example, miR-27a-3p reportedly acts as an oncogene which promotes cancer cell growth, invasion, angiogenesis, and immune evasion [24, 26]. Intriguingly, miR-27a-3p is also reported to be a tumor suppressor for repressing 17 KDa membrane-associated protein (MAP17) expression in non-small cell lung cancer [27]. In our study, we found that miR-27a-3p positively regulated LPAR6 expression and attenuated cell proliferation in luminal-type breast cancer cell line. This functional difference of miR-27a-3p may partly lie in the distinct target mRNA binding sites, for example, the non-3′ UTR region of the target gene.

There are certain limitations in this study. First, although we demonstrated the functions and potential upstream regulatory mechanism of LPAR6 in breast cancer, the downstream pathways should be investigated further. Second, miR-27a-3p was validated to be an upstream regulator of LPAR6; however, the direct regulating mechanism needs further investigation. Although further researches need to be conducted, the miR-27a-3p-LPAR6 axis is a promising therapeutic target in breast cancer.

## Conclusions

The present study provides further evidence for the expression, prognostic value, and potential mechanism of LPAR6 in breast cancer. Bioinformatics analyses reveal that LPAR6 acts as a tumor suppressor in breast cancer and may inhibit tumor progression by facilitating the formation of RB1/E2F family complexes to induce cell cycle arrest. It is also demonstrated that miR-27a-3p positively regulates LPAR6 expression, thereby attenuating cell proliferation in breast cancer. The regulation of the miR-27a-3p/LPAR6 axis would be a potential therapeutic strategy for breast cancer.

## Supplementary Information

Below is the link to the electronic supplementary material.

Supplementary file1 Fig. S1 LPAR6 expression and mutation profiles. a and b LPAR6 differentially expressed in different ethnicities and lymph node metastatic statuses (UALCAN). N0: no regional lymph node metastasis; N1: metastasis in 1–3 axillary lymph nodes; N2: metastasis in 4–9 axillary lymph nodes; N3: metastasis in 10 or more axillary lymph nodes. **p<0.01; ****p<0.0001; ns: not significant (t test). c Expression profiles of LPAR6 regarding genetic alterations and mutations spectrum. Data were obtained from cBioPortal for Cancer Genomics. d LPAR6 expression of different types of genetic alterations. Data were obtained from cBioPortal for Cancer Genomics. *p<0.05, **p<0.01, ***p<0.001, ****p<0.0001 (Kruskal–Wallis test). e Distribution of specific mutation types and mutations spectrum across breast cancer. Data were from the catalog of somatic mutations in cancer (COSMIC) database. f and g Overall survival and relapse-free survival analyses of genetic alterations in breast cancer. Data were obtained from cBioPortal for Cancer Genomics. (EPS 6465 KB)

Supplementary file2 Fig. S2 Correlation between LPAR6 expression and expression of E2F family members in tumors from METABRIC data. a–h Pearson correlation between LPAR6 expression and expression of E2F family members E2F1–8 in tumors. (EPS 6089 KB)

Supplementary file3 (DOCX 19 KB)

Supplementary file4 (DOCX 17 KB)

Supplementary file5 (DOCX 26 KB)

Supplementary file6 (DOCX 19 KB)

Supplementary file7 (DOCX 19 KB)
