# Supplementary material for: Lysophosphatidic acid receptor 6 regulated by miR-27a-3p attenuates tumor proliferation in breast cancer
Source: Clin Transl Oncol. 2021 Sep 12;24(3):503–16. doi: 10.1007/s12094-021-02704-8 (PMC8885522; doi:10.1007/s12094-021-02704-8)
Supplement: Supplementary file 3 — Supplementary file3 (DOCX 19 KB) [file 12094_2021_2704_MOESM3_ESM.docx]

| **Table S1 Knockdown of LPAR6 in MCF-7 cell line** | | | | | | |
| --- | --- | --- | --- | --- | --- | --- |
| **Group** | **Target** | **Sample** | **Ct** | **Mean** | **SD** | **p-value (t-test, relative to NC)** |
| NC | LPAR6 | NC | 26.97 | 26.99 | 0.04 |  |
|  | LPAR6 | NC | 26.97 | 26.99 | 0.04 |  |
|  | LPAR6 | NC | 27.04 | 26.99 | 0.04 |  |
|  | ACTIN | NC | 14.15 | 14.2 | 0.089 |  |
|  | ACTIN | NC | 14.15 | 14.2 | 0.089 |  |
|  | ACTIN | NC | 14.3 | 14.2 | 0.089 |  |
| si-1 | LPAR6 | si-1 | 27.93 | 28.02 | 0.159 | 0.0728 |
|  | LPAR6 | si-1 | 28.2 | 28.02 | 0.159 |  |
|  | LPAR6 | si-1 | 27.92 | 28.02 | 0.159 |  |
|  | ACTIN | si-1 | 15.07 | 15 | 0.082 |  |
|  | ACTIN | si-1 | 15.01 | 15 | 0.082 |  |
|  | ACTIN | si-1 | 14.91 | 15 | 0.082 |  |
| si-2 | LPAR6 | si-2 | 28.49 | 28.49 | 0.01 | <0.0001 |
|  | LPAR6 | si-2 | 28.48 | 28.49 | 0.01 |  |
|  | LPAR6 | si-2 | 28.5 | 28.49 | 0.01 |  |
|  | ACTIN | si-2 | 13.56 | 13.51 | 0.096 |  |
|  | ACTIN | si-2 | 13.57 | 13.51 | 0.096 |  |
|  | ACTIN | si-2 | 13.4 | 13.51 | 0.096 |  |
| si-3 | LPAR6 | si-3 | 27.41 | 27.09 | 0.333 | 0.0108 |
|  | LPAR6 | si-3 | 27.13 | 27.09 | 0.333 |  |
|  | LPAR6 | si-3 | 26.74 | 27.09 | 0.333 |  |
|  | ACTIN | si-3 | 13.54 | 13.6 | 0.05 |  |
|  | ACTIN | si-3 | 13.63 | 13.6 | 0.05 |  |
|  | ACTIN | si-3 | 13.63 | 13.6 | 0.05 |  |
