# Supplementary material for: Lysophosphatidic acid receptor 6 regulated by miR-27a-3p attenuates tumor proliferation in breast cancer
Source: Clin Transl Oncol. 2021 Sep 12;24(3):503–16. doi: 10.1007/s12094-021-02704-8 (PMC8885522; doi:10.1007/s12094-021-02704-8)
Supplement: Supplementary file 4 — Supplementary file4 (DOCX 17 KB) [file 12094_2021_2704_MOESM4_ESM.docx]

| **Table S2 Overexpression of LPAR6 in SK-BR-3 cell line** | | | | | | |
| --- | --- | --- | --- | --- | --- | --- |
| **Group** | **Target** | **Sample** | **Ct** | **Mean** | **SD** | **p-value (t-test, relative to NC)** |
| NC | LPAR6 | NC | 34.92 | 34.72 | 0.211 |  |
|  | LPAR6 | NC | 34.5 | 34.72 | 0.211 |  |
|  | LPAR6 | NC | 34.75 | 34.72 | 0.211 |  |
|  | Actin | NC | 16.14 | 16.18 | 0.035 |  |
|  | Actin | NC | 16.2 | 16.18 | 0.035 |  |
|  | Actin | NC | 16.2 | 16.18 | 0.035 |  |
| LPAR6 | LPAR6 | LPAR6 | 20.06 | 19.74 | 0.299 | <0.0001 |
|  | LPAR6 | LPAR6 | 19.47 | 19.74 | 0.299 |  |
|  | LPAR6 | LPAR6 | 19.68 | 19.74 | 0.299 |  |
|  | Actin | LPAR6 | 16.21 | 16.19 | 0.025 |  |
|  | Actin | LPAR6 | 16.19 | 16.19 | 0.025 |  |
|  | Actin | LPAR6 | 16.16 | 16.19 | 0.025 |  |
