# Supplementary material for: Lysophosphatidic acid receptor 6 regulated by miR-27a-3p attenuates tumor proliferation in breast cancer
Source: Clin Transl Oncol. 2021 Sep 12;24(3):503–16. doi: 10.1007/s12094-021-02704-8 (PMC8885522; doi:10.1007/s12094-021-02704-8)
Supplement: Supplementary file 5 — Supplementary file5 (DOCX 26 KB) [file 12094_2021_2704_MOESM5_ESM.docx]

| **Table S3 Expression of miR-27a-3p in mammary epithelial cell line and breast cancer cell lines** | | | | | | |
| --- | --- | --- | --- | --- | --- | --- |
| **Group** | **Target** | **Sample** | **Ct** | **Mean** | **SD** | **p-value (t-test, relative to NC)** |
| MCF-10A | U6 | MCF10A | 14.55 | 14.49 | 0.101 |  |
|  | U6 | MCF10A | 14.37 | 14.49 | 0.101 |  |
|  | U6 | MCF10A | 14.54 | 14.49 | 0.101 |  |
|  | miR-27a-3p | MCF10A | 20.41 | 20.36 | 0.047 |  |
|  | miR-27a-3p | MCF10A | 20.37 | 20.36 | 0.047 |  |
|  | miR-27a-3p | MCF10A | 20.31 | 20.36 | 0.047 |  |
| ZR-75-1 | U6 | ZR75-1 | 13.23 | 13.29 | 0.06 | <0.0001 |
|  | U6 | ZR75-1 | 13.35 | 13.29 | 0.06 |  |
|  | U6 | ZR75-1 | 13.3 | 13.29 | 0.06 |  |
|  | miR-27a-3p | ZR75-1 | 21.17 | 21.29 | 0.168 |  |
|  | miR-27a-3p | ZR75-1 | 21.23 | 21.29 | 0.168 |  |
|  | miR-27a-3p | ZR75-1 | 21.48 | 21.29 | 0.168 |  |
| MCF-7 | U6 | MCF7 | 13.15 | 13.31 | 0.16 | <0.0001 |
|  | U6 | MCF7 | 13.47 | 13.31 | 0.16 |  |
|  | U6 | MCF7 | 13.3 | 13.31 | 0.16 |  |
|  | miR-27a-3p | MCF7 | 22.09 | 22.03 | 0.092 |  |
|  | miR-27a-3p | MCF7 | 22.06 | 22.03 | 0.092 |  |
|  | miR-27a-3p | MCF7 | 21.92 | 22.03 | 0.092 |  |
| T47D | U6 | T47D | 13.64 | 13.74 | 0.096 | <0.0001 |
|  | U6 | T47D | 13.76 | 13.74 | 0.096 |  |
|  | U6 | T47D | 13.83 | 13.74 | 0.096 |  |
|  | miR-27a-3p | T47D | 24.29 | 24.24 | 0.037 |  |
|  | miR-27a-3p | T47D | 24.23 | 24.24 | 0.037 |  |
|  | miR-27a-3p | T47D | 24.21 | 24.24 | 0.037 |  |
| SK-BR-3 | U6 | SK-BR-3 | 13.3 | 13.36 | 0.06 | <0.0001 |
|  | U6 | SK-BR-3 | 13.35 | 13.36 | 0.06 |  |
|  | U6 | SK-BR-3 | 13.42 | 13.36 | 0.06 |  |
|  | miR-27a-3p | SK-BR-3 | 24.03 | 24.04 | 0.051 |  |
|  | miR-27a-3p | SK-BR-3 | 24.09 | 24.04 | 0.051 |  |
|  | miR-27a-3p | SK-BR-3 | 23.99 | 24.04 | 0.051 |  |
| BT549 | U6 | BT549 | 11.13 | 11.25 | 0.116 | <0.0001 |
|  | U6 | BT549 | 11.27 | 11.25 | 0.116 |  |
|  | U6 | BT549 | 11.36 | 11.25 | 0.116 |  |
|  | miR-27a-3p | BT549 | 21.56 | 21.69 | 0.117 |  |
|  | miR-27a-3p | BT549 | 21.78 | 21.69 | 0.117 |  |
|  | miR-27a-3p | BT549 | 21.73 | 21.69 | 0.117 |  |
| MDA-MB231 | U6 | MB231 | 12.93 | 13.02 | 0.08 | <0.0001 |
|  | U6 | MB231 | 13.04 | 13.02 | 0.08 |  |

**Table S3 continued**

| **Group** | **Target** | **Sample** | **Ct** | **Mean** | **SD** | **p-value (t-test, relative to NC)** |
| --- | --- | --- | --- | --- | --- | --- |
| MDA-MB231 | U6 | MB231 | 13.08 | 13.02 | 0.08 | <0.0001 |
|  | miR-27a-3p | MB231 | 21.43 | 21.42 | 0.047 |  |
|  | miR-27a-3p | MB231 | 21.46 | 21.42 | 0.047 |  |
|  | miR-27a-3p | MB231 | 21.37 | 21.42 | 0.047 |  |
| MDA-MB436 | U6 | MB436 | 13.09 | 13.48 | 0.339 | <0.0001 |
|  | U6 | MB436 | 13.7 | 13.48 | 0.339 |  |
|  | U6 | MB436 | 13.65 | 13.48 | 0.339 |  |
|  | miR-27a-3p | MB436 | 21.21 | 21.51 | 0.286 |  |
|  | miR-27a-3p | MB436 | 21.55 | 21.51 | 0.286 |  |
|  | miR-27a-3p | MB436 | 21.77 | 21.51 | 0.286 |  |
| MDA-MB468 | U6 | MB468 | 13.24 | 13.23 | 0.135 | 0.0011 |
|  | U6 | MB468 | 13.09 | 13.23 | 0.135 |  |
|  | U6 | MB468 | 13.36 | 13.23 | 0.135 |  |
|  | miR-27a-3p | MB468 | 19.98 | 20.07 | 0.271 |  |
|  | miR-27a-3p | MB468 | 19.85 | 20.07 | 0.271 |  |
|  | miR-27a-3p | MB468 | 20.37 | 20.07 | 0.271 |  |
