# Supplementary material for: Lysophosphatidic acid receptor 6 regulated by miR-27a-3p attenuates tumor proliferation in breast cancer
Source: Clin Transl Oncol. 2021 Sep 12;24(3):503–16. doi: 10.1007/s12094-021-02704-8 (PMC8885522; doi:10.1007/s12094-021-02704-8)
Supplement: Supplementary file 6 — Supplementary file6 (DOCX 19 KB) [file 12094_2021_2704_MOESM6_ESM.docx]

| **Table S4 The expression of miR-27a-3p in MCF-7 cell line treated with miR-27a-3p mimics and inhibitor** | | | | | | |
| --- | --- | --- | --- | --- | --- | --- |
| **Group** | **Target** | **Sample** | **Ct** | **Mean** | **SD** | **p-value (t-test, relative to NC)** |
| NC-mimics | miR-27a-3p | NC-mimics | 21.7 | 21.65 | 0.049 |  |
|  | miR-27a-3p | NC-mimics | 21.63 | 21.65 | 0.049 |  |
|  | miR-27a-3p | NC-mimics | 21.61 | 21.65 | 0.049 |  |
|  | U6 | NC-mimics | 12.84 | 12.76 | 0.132 |  |
|  | U6 | NC-mimics | 12.84 | 12.76 | 0.132 |  |
|  | U6 | NC-mimics | 12.61 | 12.76 | 0.132 |  |
| mimics | miR-27a-3p | mimics | 13.05 | 13.05 | 0.016 | <0.0001 |
|  | miR-27a-3p | mimics | 13.06 | 13.05 | 0.016 |  |
|  | miR-27a-3p | mimics | 13.03 | 13.05 | 0.016 |  |
|  | U6 | mimics | 13.33 | 13.42 | 0.095 |  |
|  | U6 | mimics | 13.52 | 13.42 | 0.095 |  |
|  | U6 | mimics | 13.42 | 13.42 | 0.095 |  |
| NC-inhibitor | miR-27a-3p | NC-inhibitor | 21.57 | 21.58 | 0.004 |  |
|  | miR-27a-3p | NC-inhibitor | 21.58 | 21.58 | 0.004 |  |
|  | miR-27a-3p | NC-inhibitor | 21.58 | 21.58 | 0.004 |  |
|  | U6 | NC-inhibitor | 13.22 | 13.26 | 0.114 |  |
|  | U6 | NC-inhibitor | 13.39 | 13.26 | 0.114 |  |
|  | U6 | NC-inhibitor | 13.18 | 13.26 | 0.114 |  |
| inhibitor | miR-27a-3p | inhibitor | 25.14 | 24.99 | 0.14 | <0.0001 |
|  | miR-27a-3p | inhibitor | 24.98 | 24.99 | 0.14 |  |
|  | miR-27a-3p | inhibitor | 24.86 | 24.99 | 0.14 |  |
|  | U6 | inhibitor | 13.06 | 13.1 | 0.065 |  |
|  | U6 | inhibitor | 13.07 | 13.1 | 0.065 |  |
|  | U6 | inhibitor | 13.18 | 13.1 | 0.065 |  |
