# Supplementary material for: Lysophosphatidic acid receptor 6 regulated by miR-27a-3p attenuates tumor proliferation in breast cancer
Source: Clin Transl Oncol. 2021 Sep 12;24(3):503–16. doi: 10.1007/s12094-021-02704-8 (PMC8885522; doi:10.1007/s12094-021-02704-8)
Supplement: Supplementary file 7 — Supplementary file7 (DOCX 19 KB) [file 12094_2021_2704_MOESM7_ESM.docx]

| **Table S5 The expression of LPAR6 in MCF-7 cell line treated with miR-27a-3p mimics and inhibitor** | | | | | | |
| --- | --- | --- | --- | --- | --- | --- |
| **Group** | **Target** | **Sample** | **Ct** | **Mean** | **SD** | **p-value (t-test, relative to NC)** |
| NC | LPAR6 | NC | 29.21 | 29.2 | 0.135 |  |
|  | LPAR6 | NC | 29.06 | 29.2 | 0.135 |  |
|  | LPAR6 | NC | 29.33 | 29.2 | 0.135 |  |
|  | GAPDH | NC | 14.24 | 14.25 | 0.042 |  |
|  | GAPDH | NC | 14.3 | 14.25 | 0.042 |  |
|  | GAPDH | NC | 14.22 | 14.25 | 0.042 |  |
| mimics | LPAR6 | mimics | 29.39 | 29.35 | 0.096 | 0.001 |
|  | LPAR6 | mimics | 29.25 | 29.35 | 0.096 |  |
|  | LPAR6 | mimics | 29.43 | 29.35 | 0.096 |  |
|  | GAPDH | mimics | 16.09 | 16.12 | 0.029 |  |
|  | GAPDH | mimics | 16.14 | 16.12 | 0.029 |  |
|  | GAPDH | mimics | 16.14 | 16.12 | 0.029 |  |
| NC-inhibitor | LPAR6 | NC- inhibitor | 29.44 | 29.31 | 0.186 |  |
|  | LPAR6 | NC- inhibitor | 29.4 | 29.31 | 0.186 |  |
|  | LPAR6 | NC- inhibitor | 29.1 | 29.31 | 0.186 |  |
|  | GAPDH | NC- inhibitor | 15.06 | 15.04 | 0.021 |  |
|  | GAPDH | NC- inhibitor | 15.02 | 15.04 | 0.021 |  |
|  | GAPDH | NC- inhibitor | 15.05 | 15.04 | 0.021 |  |
| inhibitor | LPAR6 | inhibitor | 29.74 | 29.71 | 0.079 | 0.0031 |
|  | LPAR6 | inhibitor | 29.77 | 29.71 | 0.079 |  |
|  | LPAR6 | inhibitor | 29.62 | 29.71 | 0.079 |  |
|  | GAPDH | inhibitor | 14.39 | 14.4 | 0.095 |  |
|  | GAPDH | inhibitor | 14.5 | 14.4 | 0.095 |  |
|  | GAPDH | inhibitor | 14.31 | 14.4 | 0.095 |  |
